# Mitochondrial genome of the North African Sahara Honeybee, *Apis mellifera sahariensis* (Hymenoptera: Apidae)

**DOI:** 10.1080/23802359.2017.1365647

**Published:** 2017-08-22

**Authors:** Nizar Haddad, Noureddine Adjlane, Wahida Loucif-Ayad, Abhinandita Dash, Naganeeswaran S., Balaji Rajashekar, Kosai Al-Nakeeb, Thomas Sicheritz-Ponten

**Affiliations:** a Bee Research Department, National Center for Agriculture Research and Extension, Baq’a, Jordan;; b Faculty of Science, Department of Biology, M’hamed Bougara University of Boumerdes, ENS Kouba, Algiers, Algeria;; c Faculty of Science, Laboratory of Applied Animal Biology, University Badji-Mokhtar, Annaba, Algeria;; d Genotypic Technology Private Limited, Bangalore, India;; e Department of Bio and Health Informatics, Technical University of Denmark, Lyngby, Denmark

**Keywords:** *Apis mellifera sahariensis*, genome sequence, Sahara bee, mitogenome

## Abstract

We present the complete mitochondrial genome of honey bee subspecies, *Apis mellifera sahariensis* (Apidae) belonging to the African lineage. The assembled circular genome has a length of 16,569 bp which comprises 13 protein coding genes, 22 transfer RNA genes, two ribosomal RNA genes, and AT rich region.

*Apis mellifera sahariensis* is a subspecies of honeybee (*Apis mellifera*) belonging to the African lineage and is found in the oases of the Sahara to the south of the Atlas Mountains, Aïn-Sefra, Bechar, Algeria (32°45′22.8″N, 0°34′40.092″W) (Baldensperger 1924). It has the ability to adapt to extreme conditions like temperatures in Saharan zones ranging from −10 °C to over 50 °C to drought conditions (Adjlane et al. 2016) and high altitudes (Haccour 1960). In the present study, we report the first complete mitochondrial genome of *Apis mellifera sahariensis*, which will enhance our knowledge on *Apis* mitogenomes and phylogeny, the previous studies of *Apis mellifera intermissa* (Peng et al. 2014) and *Apis mellifera syriaca* (Haddad 2015) from the Middle East North Africa Region, will help further understanding the genetic relation between these bees.

The draft genome of the *Apis mellifera sahariensis* was sequenced using Illumina HiSeq platform (150 bp paired-end chemistry) at Genotypic Technology Pvt. Ltd. (Bangalore, India). We assembled a subset of the generated raw reads from the mitochondrial genome and annotated the assembly with the MITOS webserver (Bernt et al. 2013). Evolutionary analysis of the mitochondrial genome was done using the PAUP software (Swofford 2003).

In this report, we have presented the complete circular mitogenome of *Apis mellifera sahariensis* with the a total length of 16,569 bp. The assembled genome quality was substantiated with an average vertical read depth of 22,410 bp per position. Nucleotide composition in the assembled mitogenome comprises A = 6885 bp (41.55%), T = 7165 bp (43.24%), G = 1595 bp (9.63%), C = 924 bp (5.58%) with an overall AT-rich percentage of 84.80%. A total of 37 genes which include mitochondrial protein coding genes for oxidative phosphorylation (atp6, atp8, cob, cox1, cox2, cox3, nad1, nad2, nad3, nad4, nad4l, nad5, and nad6) along with 22 tRNA genes as well as genes for the large and small ribosomal RNAs (rrnL and rrnS) were annotated. Homology search of *Apis mellifera sahariensis* against available *Apis* mitogenomes resulted in high homology and similar gene arrangement. Phylogenetic analysis showed high similarity of *Apis mellifera sahariensis* with *Apis mellifera intermissa* (KM458618) (Figure 1).

**Figure 1.**
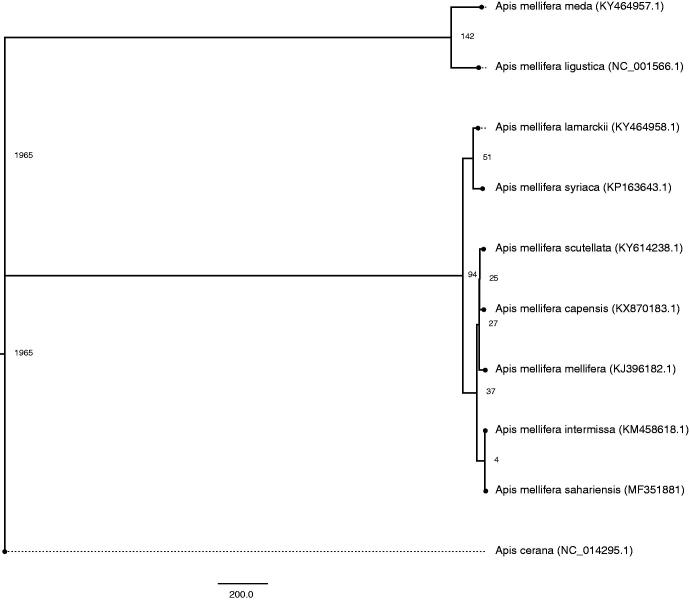
The phylogenetic tree was created using the parsimony criterion on the ungapped sequences of a multiple sequence alignment using Clustal Omega on available *Apis mellifera* sub-species.

## Nucleotide sequence accession numbers

The mitochondrial genome sequence of *Apis mellifera sahariensis* has been submitted in NCBI GenBank under the accession no. MF351881.

## References

[CIT0002] Adjlane N, Dainat B, Dietemann V, Gauthier L. 2016 Atypical viral and parasitic pattern in Algerian honey bee subspecies *Apis mellifera intermissa* and *A. m. sahariensis*. Apidologie. 47:631–641.

[CIT0004] Baldensperger PJ. 1924 North African Bees, II. Bee World. 5:189–190.

[CIT0005] Bernt M, Donath A, Externbrink F, Florentz C, Fritzsch G, Jühling F, Middendorf M, Pütz J, Stadler PF. 2013 MITOS: improved de novo metazoan mitochondrial genome annotation. Mol Phylogenet Evol. 69:313–319. 22982435 10.1016/j.ympev.2012.08.023

[CIT0007] Haccour P. 1960 Recherche sur la race d’abeille saharienne au Maroc. Bull Soc Sci Nat Phys Maroc. 6:96–98.

[CIT0008] Haddad N. 2015 Mitochondrial genome of the Levant Region honeybee, *Apis mellifera syriaca* (Hymenoptera: Apidae). Mitochondrial DNA J. 27:4046–4068. 10.3109/19401736.2014.1003846 25633178

[CIT0011] Peng H, Lu ZX, Haddad N, Noureddine A, Loucif-Ayad W, Wang YZ, Zhao RB, Zhang AL, Guan X, Zhang HX, et al 2014 Complete mitochondrial genome of the Algerian honeybee, *Apis mellifera intermissa* (Hymenoptera: Apidae). Mitochondrial DNA. 27:1791–1792. 25259457 10.3109/19401736.2014.963815

[CIT0013] Swofford DL. 2003 PAUP*. Phylogenetic analysis using parsimony (*and other methods). Version 4. Sunderland, MA: Sinauer Associates.

